# Essai Sur La Philosophie Medicale. M. Bouillaud on Medical Philosophy

**Published:** 1837-04-01

**Authors:** 


### ESSAI SUR LA PHILOSOPHIE MEDICALE. Par *M. Bouillaud.* Paris, 1836.

ANOTHER work from the pen of M. Bouillaud! This ever active and most enterprising Professor appears to allow no respite to the labours of authorship, and certainly not one moment of truce to the opponents of those doctrines of which he is such an able champion. In the course of the last eighteen months he has published, in addition to the present volume, a large treatise on the diseases of the heart, and another, of smaller dimensions indeed, on rheumatism. He is physician to one of the largest hospitals in Paris, La Charité, is Professor of Medicine to the Faculty, and is a frequent contributor to the medical periodicals, especially to the Journal Hebdomadaire, of which he was for many years the sole editor. At an earlier period of his career, he published some interesting researches on typhus fever—the entero-mesenteric fever of his nosology—and he was, we believe, the joint editor of M. Bertin's work on the diseases of the heart. Although still only a middle-aged man, he has thus achieved for himself an eminently meritorious reputation, as one of the most indefatigable, if not one of the most cautious and philosophical, physicians in the metropolis of France. Having attached himself to the school of M. Broussais, he devotes every power of his mind, and every opportunity of public speaking and of public writing to confirm and illustrate the doctrines of the great medical Reformer of the nineteenth century; and certainly, if unwearied zeal, intrepid energy, and a keen lively imagination, in union too with very considerable talents for research and for forcible description, could effect his wishes, then indeed M. Broussais might fairly "seize and keep the sceptre of medicine," which our author so loyally awards to him.

As might be supposed, all the writings of M. Bouillaud are tinctured with, we should rather say, steeped in, the very spirit of Broussaism; and it is not therefore at all difficult to acquaint the reader with their general scope and tendency. There are three or four leading doctrines, which lie at the bottom of all his writings, whether these be in the shape of lectures, or of formal treatises. These are—the connexion, or rather the dependence, of typhus with a diseased state of the mucous membrane, and especially of the mucous glands, of the bowels;—the vehemently inflammatory nature of acute rheumatism, and the almost invariable co-existence of pericarditis or endocarditis along with it—the origin of almost all organic diseases in chronic phlegmasia of the parts affected—and the extreme rarity of purely nervous or functional diseases.*

The great therapeutic indication on which M. Bouillaud insists, in the treatment of all pyrexiæ, is active and repeated bleeding. He is not satisfied with minute *doses* of blood: these, he says, are totally inefficacious. His practice is very different; he strives to strangle (juguler) the foe at the outset, and for this purpose he repeats the bleeding "coup sur coup," as long as there is any vigorous vitality in the mischief.

M. Bouillaud unquestionably deserves, to a certain extent, credit for having strongly recommended, and for having partially introduced into French practice, a more vigorous and decided plan of treatment in active inflamma-

---

* In noticing the cliniques of M. Bouillaud in our Foreign Periscope, we have repeatedly taken occasion to allude to the almost total absence of any of the "neuroses" in the wards of La Charité. The reports of his lectures there are altogether occupied with cases of fever, pneumonia, pleuritis, and other phlegmasiæ, disease of the heart and large bloodvessels, rheumatism, and a few other cognate maladies. We much fear that students educated in such a school must be ill prepared to encounter many of the perplexing casualties of private practice. The Protean forms of hysteria, dyspepsia, neuralgia, &c. constitute, at least in this country, the staple commodity of practice among the wealthier classes of the community.

tion, than his countrymen have hitherto been willing to adopt. Their remedial measures have been generally far too feeble and inert in the more severe forms of pneumonia, pleuritis, rheumatism, &c. and we are quite confident that if the results of their hospital experience in such diseases were compared with those in British hospitals, they would be found to be greatly less successful. But while we say this, we are very far from being disciples of M. Bouillaud, or of those physicians who can see nothing else in typhus fever, (in cholera also, we believe,) in rheumatism, in erysipelas, and other exanthemata, than merely a local inflammation, and who most perniciously imagine that the only indication of treatment in those diseases is to draw blood, and to deplete the system in other ways. In our author's own words, "the grand triumph of medical science in the present century has been the localization of many diseases which were formerly considered essentially affecting the whole and every part of the frame; and the mighty champion of this all-important change is the immortal Broussais." This is the ever-recurring theme of our author's discourses; and certainly of none more than of the one now under review. But he is too sharp sighted not to perceive the weak points of this pet doctrine, and not to see the necessity of admitting that the *fluids* as well as the *solids* may primarily become altered in some diseases. He alludes to the phenomena of scurvy; and assuredly he could not specify one in the nosological catalogue more appropriate, and one which more unexceptionably illustrates the truth of a cautious scientific humoral pathology. The changes in the state of the blood in certain fevers are almost as conspicuous as in scurvy; and we have no doubt but that the successful treatment of these will be found to depend upon a due attention being paid to obviate and rectify those, hitherto ill-understood, morbid humoral changes.

But it is high time now to introduce our readers to an acquaintance with the "Philosophie Medicale."

Before entering upon a short review of its contents, we must enter our protest against the mode of authorship pursued by M. Bouillaud. He writes with fluency, ease and force; his ideas come crowding in upon him, and he seems to be never at a loss for energetic and vivacious language to express them. But then there is no method, no labour of arrangement, no regular plan of reasoning throughout the work. The reader is every now and then taken by surprise, from the suddenness with which some new topic is introduced, and next page perhaps deserted; then again brought forward, to be as quickly dispatched; and thus the general impression left on his mind, when he comes to the end, is, that the book is a patchwork of most inconclusive and unsatisfactory discourses.

We can quite picture to ourselves M. Bouillaud at his desk. Bold, rapid, and very clever, once that the pen is in his hand—on it goes without stop or restraint. Like his ardent colleague, M. Velpeau, he may make a boast of writing a book in the course of a week or two "currente calamo." The boast is so truly *à la Française* that we might admit its plea, as a *petitio favoris*, were we not called upon, in our reviewing capacity, to read, peruse, and try to digest the wearisome pages of so many tedious tomes of such a writer as M. Bouillaud.

We are quite confident that if any one of the works of such an author as M. Bouillaud was shorn of its unnecessary pages, and its useful contents only were presented to the public, they would not occupy one quarter of their present bulk. We are no disciples of Dr. Hahnemann in the practice of medicine; but most assuredly we should recommend the precepts about infinitesimal doses to some of the authors of the present day.

These prefatory remarks may possibly be of use to those who peruse for themselves the "Philosophie Medicale" of M. Bouillaud; and they will prepare our own readers for the short review which we now propose to take of its contents. The first division of the work is occupied with a historical sketch of medicine. The author takes a rapid, and, as may be supposed, a very incomplete review of the leading schools or systems of physic, which have prevailed from the time of Hippocrates till towards the close of the 18th century, when those two bright luminaries, Bichat and Pinel, arose and vindicated for their country the pre-eminent claim of being the birth-place of modern medical science.

They may be justly considered as the founders of systematic or philosophical medicine in France, and as the primary movers of all those great changes which have been effected by their countrymen since. It is certainly a curious and interesting fact in the history of our profession, that, before their time, France had not given birth to any school or sect of physicians, who carried much influence or had gained many followers in foreign countries;* and that while Germany, Holland, Britain, and Italy could boast of their Hallers, their Boerhaaves, their Sydenhams, and their Morgagnis, little was heard of that land, which within the last 50 years has occupied by far the most conspicuous station in the career of medical improvement, and can now proudly point to at least a score of glorious names in medicine.

To Bichat and Pinel is unquestionably due the honour of having vindicated the medical eminence of their country; and none assuredly are more willing than ourselves to admit the many and most important benefits which they, and their successors up to the present day, have conferred on medical science.

We cannot, indeed, but regret that the French are still so very ignorant of what has been done, and what is doing in other countries. M. Bouillaud, for example, appears to be entirely ignorant of English medical literature; and in this work, which is somewhat boastfully denominated an Essay on Philosophical Medicine, it will scarcely be believed that not a single reference is made to any British physician, save to our distinguished Sydenham, and who, perhaps, has to thank only his Roman garb for this casual notice. The writings of Hunter, Heberden, and of Cullen are, we should think, quite unknown to him; and perhaps, therefore, we should not be much surprised, that even the ordinary practice of British physicians in acute internal inflammations, we allude to the use of large and repeated bleedings, has never

* We ought, perhaps, to make an exception in favour of Sauvages, who was one of the professors, during the early part of last century, in the medical school of Montpelier, which long held the first rank in France. His methodical nosology has been esteemed a work of very considerable merit, and may be considered as having been the precursor of Cullen's better-known system; but it never commanded, we should think, much influence beyond the sphere of the Professor's own pupils.

been heard of by the French Professor. He always mentions such practice, as if it was an undisputed *discovery* of his own. But we shall recur to this topic afterwards; and we shall, therefore, now proceed, without further delay, to notice his comments on Bichat and Pinel's physiological and pathological doctrines, and on the influence which these doctrines have had on the present philosophy of medicine.

The basis and essential principle of Bichat's system may be briefly stated to be, the referring of all the actions, movements, and effects of living bodies to the operation of certain Vital powers, which are quite distinct from the laws of inanimate matter. Of these powers he made a twinfold division, denominating them by the names of Contractility, and Sensibility. He was strictly and essentially a vitalist in all his speculations; and in the retrospective view, which he takes of the different systems which had preceded him, he mentions with especial praise those of Van Helmont and of Stahl, who, rejecting the chemical and mechanical hypotheses of their cotemporaries, had the sagacity to refer all the operations of the living economy to the presiding and controlling influence of a vital principle, which they denominated Archæus, or Anima. Barthez and Chaussier, his countrymen, adopted with certain modifications the views of Van Helmont and Stahl, and with considerable ingenuity had explained the functions of the body by attributing them all to the action of a vital force. But Bichat perceived the fallacy of thus attributing all the operations of life to a single, undefined principle or law, (whatever name we choose to give it), whose very existence is purely abstract and ideal. A spirit of metaphysical speculation had thus become blended with physical researches. The assumed presiding principle of living matter had been invested with powers and attributes akin to those of reasoning; and hence a vague and jargonish language had been introduced into most works on medical science. Bichat shewed that the only legitimate aim of the physiologist's enquiries is, to study the properties or functions of living bodies, in health and in disease; and to endeavour to find out their mutual dependencies, the phenomena which they exhibit, the action of external agents upon them, and the uniformity or irregularity of their operations.

We have already stated that, he grouped all the vital actions under the two great powers or principles of contractility and sensibility. By contractility he meant that power, in virtue of which the solid parts of a living body are capable of contracting, or of being excited to certain movements, either from the direct application of stimuli to their fibres, or from the interposed operation of the brain upon them.

He described two forms of contractility—the organic, and the animal. The organic contractility appears to be exercised more or less independently of the action of the brain. All that is necessary for its being called into action, is merely the application of a *stimulus* directly to the part. The phenomena which Bichat referred to the operation of organic contractility are sometimes manifest and appreciable by the senses; at other times they are obscure, latent, and are rather assumed than proved to take place. As examples of the former, we may mention the puckering and shrinking of the skin from cold, the vermicular motion of the intestines, the expulsory action of the urinary bladder, uterus, the contraction of the heart, &c. These and such like phenomena Bichat referred to what he called the "sensible organic contractility" of living bodies. It very nearly corresponds to the "irritability" of Haller. It is well known to exist for some time after the death of the individual; the period of its persistence varying much in different parts, and in different tribes of animals.

The other set of actions—those, namely, which cannot be perceived by our senses, but which, we infer, must take place in consequence of the results produced—is exemplified in the movements of the fluids in the capillary vessels, in the various secretory and excretory tubes, in the absorbents, &c. Such actions are referred to the "insensible organic contractility" of these parts. This power or property belongs to all living bodies without exception; to vegetables as well as to animals. By some authors it has been denominated "tonicity"—by others "fibrillar contractility."

Very different from either form of organic contractility is the animal, or, what has been at other times called, the cerebral contractility. All the voluntary movements, and many of those which are partly voluntary and partly involuntary, such as the actions of the abdominal and respiratory muscles in the expulsion of the intestinal, urinary, and uterine contents, &c. are to be arranged here.

The other great power also of living bodies—Sensibility—Bichat divided into two forms, animal, and organic sensibility. The former is accompanied with actual feeling or consciousness; the latter is not, and implies only that living bodies are susceptible of being acted upon by their appropriate stimuli, without the consciousness or the will of the individual. The language adopted by Bichat is certainly very faulty, and apt to mislead the reader. When we speak of sensibility, the mind very naturally associates it with the idea of sensation. Now the organic sensibility of Bichat's system is used to denote the very absence of this power or property. The French physiologists of late have, therefore, very properly substituted the term "impressionability;" appropriating that of "sensibility" to the animal sensibility of Bichat.

To these two general properties or principles now described, Bichat attributed all the operations of living animal bodies; and he ingeniously attempted to explain the proximate causes or intimate nature of diseases by referring them to some particular lesions or irregular actions of these properties. We shall extract one passage from his great work, as a specimen of his method: "Dans les maladies nerveuses, c'est *la sensibilité animale* qui joue un si grand role; les convulsions et les paralysies consistent dans une augmentation, ou une diminution de la *contractilité animale;* toute la classe des fievres, toutes les affections gastriques, &c. sont une trouble manifeste dans la *contractilité organique.* Les tumeurs de nature diverse, les exhalations augmentées, le marasme, &c. supposent toutes un trouble dans la *sensibilité organique* et dans la *contractilité insensible* correspondante."

Presuming that the vital properties or functions reside essentially in the solids, and that all the phenomena of disease are only the outward signs or manifestations of some disturbance of these properties, Bichat was obliged, even in spite of many powerful objections, to acknowledge himself to be strictly and truly a solidist. And yet he saw his error.

"Do not, however, believe (says he, addressing the reader), that the fluids are of no import in disease; very often they contain and convey the morbid germ, the morbific matter. It is by the fluids, destined as they are for the nutrition of the organs, that disease is often conveyed to the solid parts: and, on the other hand, it is by the excretory fluids, that disease is often eliminated from the system. Physicians have, it is true, greatly exaggerated the influence of morbific humours in the generation of disease; but we should not, therefore, fall into the opposite error, and dismiss the humoral pathology wholly from our reasonings. It is evident that we should always well discriminate between a disease itself, or, more correctly speaking, the *ensemble* of the symptoms which characterise its presence, and the principle or cause which produces it. Almost all the symptoms of a disease are manifested by the solids; but the cause of it may reside wholly or partially in some of the fluids."

There is great physiological acuteness, and practical wisdom in these remarks. It would be interesting to trace at considerable length the ideas of the immortal author on general pathology; but, at the present time, we cannot spare room for this enquiry. There is, however, one short sentence, which from its important truth deserves especially to be never forgotten:—" Toute theorie exclusive de solidisme, ou d'humorisme est un *contre-sens pathologique*, comme une theorie dans laquelle on mettrait uniquement en jeu les solides ou les fluides en serait un physiologique." Although unwilling to assert that the fluids during life are in themselves possessed of vital properties, Bichat did not hesitate to admit that they might become diseased, and thus might be the cause of disease throughout the system, in consequence of internal molecular changes. Alluding to the changes of the blood in some diseases, he says; " Dernièrement nous avons trouvé, au lieu de sang noir abdominal, une véritable sanie grisatre qui remplissait toutes les divisions de la veine splenique, le tronc de la veine porte, et toutes ses branches hepatiques. Cette sanie n'etait pas un effet cadaverique, et le sang avait circulé, sinon altéré, au moins bien different de son etat naturel, et réellement decomposé."

These extracts will suffice to shew that Bichat was not, as some have alleged, exclusively a solidist in his ætiological speculations, even although he professed himself to be so. He was far too enlightened not to perceive the error of studying one set only of living phenomena, to the exclusion of another equally important one. The great excellence, however, of Bichat consisted less in exploring the intimate nature of any class or classes of disease, than in discovering and most ably elucidating the relations of resemblance and of identity between the diseases of distant parts.

It was he who first pointed out, or at least who first established the similitude in point of structure, functions, and morbid changes of certain tissues, such as the mucous, the serous, the fibrous, and so forth, in whatever part of the body they happen to exist. His own words are so excellent, that we shall give them in the original.

" Puisque chaque tissu organisé a une disposition partout uniforme; puisque, qu'elle que soit sa situation, il a la meme structure, les memes proprietés, il est evident que ses maladies doivent etre partout les memes. Que le tissu sereux appartient au cerveau par l'arachnoide, au poumon par la plevre, au cœur par le pericarde, aux viscères gastriques par le peritoine, partout il s'enflamme de la meme manière, partout les hydropisies arrivent uniformement, et partout il est sujet à une espece d'eruption de petits tubercules blanchatres, comme miliaires. Quelque soit aussi l'organe que revete le tissu muqueux, ses affections portent en general le meme caractère, et n'offrent point d'autres variétés, que celles qui proviennent des variétés de structure."

Here we have the essence, the pith and marrow of Bichat's great discoveries.

His doctrines, when first announced, were received with the greatest enthusiasm, and adopted with the most implicit submission. Such is the homage which an original and brilliant genius will ever command. But it was speedily discovered that the system of Bichat, like every other which is founded on a pure and exclusive vitalism, was insufficient to explain many of the operations of the body, at least in disease. A few facts will readily prove this to be the case.

Take for example some of the diseased states of the heart; such as contractions of the valves, ossification of the arteries, &c. It is obvious that to attempt to explain the origin or proximate cause of these changes by referring them to any lesion of contractility or of sensibility, is, if not absolutely absurd, at least without any profitable meaning. Then, too, what are we to make of those decompositions and combinations, those analyses and syntheses, in short, those reactions of every sort, which are going on at all times in the laboratory of a living body? Can sensibility and contractility explain the chemical phenomena of respiration, or of digestion? The universal action of secretion must be, in some measure, connected with the play of certain affinities, hitherto, we confess, undiscovered, but not the less probable. As yet little progress has been made in what may be aptly called "living chemistry;" but we are quite confident that it is to this science, more than to any other, that we must look for any great discoveries in ætiological medicine.

The system of Bichat was certainly a great improvement upon the vitalist systems, which had preceded it. By breaking down, by decomposing, by analysing the complex principle, known under the varying denominations of Nature, enormon, vis insita, impetum faciens, archæus, anima, force vitale, &c. the great French physiologist, divested his favorite science of much of its metaphysical obscurity; and although, we must confess, he has committed a great error in excluding all reference to mechanical and chemical actions in his analysis of physiological phenomena, let us never forget that it was he, who laid the foundation of general anatomy—the only true basis of a rational classification of vital phenomena.

In his own emphatic language, he has tried to do for physiological science, what Newton had done for physical science; to point out and illustrate that secret of creating power—"la simplicité des causes reunie à la multiplicité des effets." The attempt indeed was a noble one, and its execution truly wonderful, when we think that the mighty architect was not more than 30 years of age at the period of his death.

Among the numerous rivals and successors of Bichat, no one was so well qualified to illustrate and to extend his discoveries, as he, "dans les mains duquel devait bientôt tomber le sceptre de la chirurgie moderne." Dupuytren himself published but very little on anatomical and physiological subjects; but his admirable views and doctrines have been made known by the writings of several of his pupils.

Among these, Marandel's Essai sur les Irritations, published in 1808, Cruveilhier's work on Pathological Anatomy, and Roche and Sanson's excellent treatise on Practical Medicine and Surgery, deserve especial notice.

Having thus briefly alluded to the labours of Bichat, our attention is next directed to his great contemporary, the philosophic Pinel. France is justly proud of this eminent physician. He contributed largely to raise the reputation of the School of Paris to its present distinguished rank.

In 1798, the first edition of the Nosographie Philosophique—a book which for nearly twenty years was "le code, l'evangile de l'Europe medicale"—was published. Six editions of this voluminous work were exhausted in that period; and in spite of many defects and not a few errors, M. Bouillaud characterises it as "le plus beau monument qui eut encore été elevé à la medecine," whether we regard it as a philosophical and systematic review of medical science, or as a descriptive treatise of diseases. The varied erudition of the author, his long and extensive practice, his great experience in clinical teaching, and the philosophical bias and discipline of his mind, had quite formed him to be the founder of a system, or school of physic.

In the Nosographie Philosophique, all diseases are arranged under five classes—fevers, inflammations, hæmorrhages, neuroses, and organic lesions. Pinel was well aware how exceedingly difficult it is to reduce any groupe of diseases to strict, or well-defined characters or rules. We find therefore that, in the last edition of the Nosographie, published in 1818, (and therefore subsequent by some years to Broussais' Examen) he alludes to the classification of fevers in the following terms: "La division nosologique des fievres m'a paru de plus en plus remplie de difficultés, à mesure que j'ai cherché à la rendre plus exacte et plus complete. Si on veut etre severe dans ses jugements, la doctrine des fievres, telle que je l'ai exposée jusqu'ici, serait sans doute, dans les circonstances actuelles, susceptible de quelques changements, ou du moins enoncée sur quelques points avec les caracteres du doute, si les faits particuliers, sur lesquels portent les assertions generales, etaient plus precis, et leur methode descriptive plus perfectionnée;" and before treating of the last class, that of organic lesions, he has very justly remarked that "elles offrent tant de disparate qu'on peut à peine esperer de les soumettre à un ordre regulier de distribution." It is especially worthy of notice, in reference to the subject of fevers, that there is strong reason to believe that Pinel did not in truth believe in their *essentiality*, at least to the full extent which a reader might be led to suppose from his work. He was well acquainted with the description which Rœderer and Wagler had given of the morbid appearances in the intestines, found in the bodies of those who had died of a mucous fever at Gottingen in 1761; and M. Rostan, in his late Cours de Medecine Clinique, expressly assures us that Pinel followed rather the advice of some friends, than the convictions of his own judgment, in not classifying the groupe of fevers among the phlegmasiæ. The words of M. Rostan are these:—"Le celebre auteur de la Nosographie Philosophique est le premier qui ait senti le vague qui regnait dans l'histoire des fievres continues. Aussi, dans ses premiers travaux (les premiers conceptions chez les hommes de genie sont presque toujours les meilleures), avait-il revoque en doute l'existence de ces fievres; et, si plus tard vaincu par de timides conseils, craignant les animosités medicales, it crut devoir en tracer le tableau dans ses ouvrages, au moins fit-il de grands efforts pour les *localiser*."

The Nosographie was, as might be expected, exposed to numerous attacks from various quarters. The observations of M. Prost in his "Medecine Eclairée par l'Ouverture des Corps," and the more memorable monograph of MM. Petit and Serres on the "fievre entero-mesenterique," sapped the foundations of M. Pinel's doctrines on fevers; and then the beautiful researches of M. Broussais on chronic phlegmasiæ, exposed the feeble and faulty arrangement of his fifth division—that of organic lesions. It was in 1816 that the last-named author first directly assailed the doctrines of Pinel on the nature of fevers. M. Pinel, in the last edition of his work, published two years afterwards, alludes to this attack, in a passage which deserves to be recorded.

"It is well known that, since the last edition of the Nosography was published, attempts have been made to introduce a new system of medical philosophy. I do not feel at all inclined to enter at present into any discussion of its merits. I will only remark that it appears to be a painful restraint for certain *effervescent* authors to keep within moderate bounds; they perceive, and perhaps may discover a few isolated and occasional facts; their fancy becomes quite heated in consequence, and at length they fairly believe that they are able to overturn the whole system of medical knowledge, and to establish certain exclusive doctrines of their own.

Those minds, which are but superficially informed, and not accustomed to the slow and gradual progress of inductive science, are readily seduced by such dogmatists. It is thus that the class of primary or essential fevers has been of late years removed into that of the phlegmasiæ, and that it has been attempted to exclude altogether from medical reasoning, the words, 'adynamic' and 'ataxic.'"

We have already alluded to M. Prost's work, La Medecine eclairée par l'Ouverture des Corps.

It appears to be much less generally known than it deserves to be; for even MM. Petit and Serres, although writing on the same subject only a few years afterwards, do not notice it. M. Prost had unquestionably anticipated M. Broussais in many of the doctrines which have been attributed to him, as their author. In the preface to La Medecine, &c. M. Prost states that he minutely dissected more than 400 cases of disease—each dissection occupying him many hours, and some even an entire day—before he resolved to publish his observations.

His great object was to prove that every form of fever is associated with some internal inflammation, whether of the bloodvessels, or of the thoracic, and, more especially, of the abdominal viscera. Treating of bilious fevers, he distinctly announces his opinions in these words:—"Les symptomes ataxiques sont l'effet de la phlogose intestinale. Les symptomes adynamiques sont relatifs à la termination de l'inflammation de la tunique muqueuse des intestins par gangrene, et à l'eloignement plus ou moins rapide du sang des vaissaux dans lesquels il abondait d'abord;" and in another passage he repeats the same sentiment: "Le cerveau peut sans doute éprouver des disordres provenant des phlogoses, qui ont lieu dans sa substance ou ses membranes; mais ce n'est point à ces affections que sont dues les fievres ataxiques: l'alteration organique, qui leur donne lieu, consiste dans l'inflammation de la membrane interne des intestins, avec ou sans excoriations." He states that he had examined upwards of 200 fatal cases of ataxic fever, and that, in every one, the intestinal mucous membrane was found more or less inflamed.

It is very strange that Pinel should never have noticed these observations of M. Prost, in any of the later editions of his Nosographie; and it is still more strange and worthy of notice, that Broussais himself, in the first edition of his Histoire des Phlegmasies Chroniques, should have impugned their accuracy. After having mentioned the opinion of M. Prost, as to the influence of gastro-enteritis on the production of Ataxic fever, he adds, "j'ai trop souvent rencontré cette membrane en bon etat, à la suite des typhus les plus malins; j'en ai vu un trop grande nombre s'ameliorer par l'emploi des stimulants les plus energetiques, pour partager l'opinion de ce medecin sur la cause de la fievre ataxique." It was not, till the third* edition of the Histoire was published, that the great reformer announced his recantation of these opinions, and adopted the very creed which a few years before he had assailed. Such is the fluctuation of medical doctrine!

In 1813, nine years after the publication of M. Prost's observations, appeared the famous treatise on Entero-Mensenteric Fever, by MM. Petit and Serres. These authors mention, how much they were misled, at first, as to the true nature and proper treatment of the fever, by regarding it as of the Adynamic or Ataxic order; and even after they understood its pathology, how apt they were to overlook the abdominal mischief, in consequence of the great obscurity, and sometimes of the total absence of the local symptoms. The morbid appearances, which were discovered by them on dissection in almost every fatal case, were enlargement and ulceration of the glandulæ Peyeri in the lower part of the ileum and head of the colon, and enlargement and often softening of the adjoining mesenteric glands. The mucous membrane of the other parts of the intestines was usually but little, if at all, diseased; and the rest of the abdominal viscera exhibited none of the usual signs of inflammatory action.

In one case only was the entero-mesenteric fever complicated with genuine enteritis. But it appears that their improved pathological knowledge did not lead MM. Petit and Serres, in M. Bouillaud's opinion, to take a correct view of the disease, or to treat it properly. The abdominal lesions, it must be known, were not regarded by them as resulting from active inflammation; —their own expression is, "la lesion de l'intestin et du mesentere n'etait pas inflammatoire, il est vrai"—and therefore so far from recommending an antiphlogistic treatment, they frequently used, with the happiest effects, bark, æther, camphor, wine, &c.

All this is very different from the doctrines and practice of Broussais† and his disciples. M. Bouillaud warmly supports all his views, and seems to regard him as an oracle of truth on all occasions. Among other terms of eulogium bestowed upon the founder of the so-called physiological system, he calls him "the Messiah destined to accomplish the regeneration of medicine!!"

---

* This fact is certainly not generally known, in this country at least. To our minds it affords strong presumptive proof, that Broussais himself has pushed his doctrines a great deal too far. None is so apt to become a bigot, as a converted infidel.—*Rev.*

† The vivacity of our author is always ingenious and sometimes very amusing. Alluding to the date of the publication of the Examen in 1816, he states that Broussais had announced and unfolded his peculiar system two years before, upon his return with the army to Paris, and adds, "Sans la grande catastrophe de 1814, qui sait si jamais M. Broussais eut accompli sa glorieuse destinée de reformateur de la medecine?"

It is almost unnecessary to state his peculiar doctrines.

The non-essentiality of fevers, the localisation of them in the mucous membrane of the digestive tube, and the dependence of almost all organic lesions upon chronic inflammation of the part affected—these are the fundamental and leading precepts of Broussais' system. That M. Broussais is truly and essentially a solidist in most of his physiological and pathological views is not to be denied; but it is unfair to assert that he has rejected altogether the influence of the fluids in accounting for the phenomena of diseases.

In treating, for example, of scurvy, (which Pinel arranges along with syphilis, gangrene, cancer, dropsy, diabetes, worms—such an olla podrida of dissimilarities!) M. Broussais expressly states that the disease commences in a corruption, or poisoned state of the blood itself: "le scorbut est veritablement une maladie humorale; et quoiqu'en puissent dire les vitalistes exclusifs et les Brownians, elle n'est pas le pur et simple effet de la debilité generale. Il est donc des cas, ou les maladies peuvent commencer par les fluides, et c'est alors par les fluides qu'il faut les attaquer."*

The doctrines of Broussais have been unreservedly adopted by a large number of his countrymen. In foreign countries, although they have had, we must confess, the beneficial effect of directing the attention of physicians in fevers to local complications, among which the most frequent and most important is certainly that of gastro-enteric inflammation, they have never met with any very cordial reception. We are not aware of a single well known author in our own country, who can be justly considered a Broussaist. In France, too, some of the ablest physicians have never renounced the doctrine of the essentiality, and idiopathic nature of continued fevers. We shall briefly notice the opinions of a few on either side of the question.

M. Andral had, in the first edition of his Clinique Medicale, treated of fevers in a class by themselves; but in the second and third editions of his work, he has distributed them among the phlegmasiæ, either of the abdominal viscera, or of the nervous centres. "Depuis la publication," says this very cautious and most accurate pathologist, "de l'Examen des doctrines, dont on connoit la haute prudence, de nombreux travaux sont venus appuyer la doctrine de la localisation des fievres."

M. Rostan, after alluding to the attempts of some physicians to banish essential fevers from the nosological catalogue, thus expresses himself: "Il etait reservé à M. Broussais de combattre avec avantage ces croyances de l'ancienne medecine: c'est une justice que nous aimons à lui rendre, et la posterité se plaira sans doute à reconnoitre un service si eminent."

M. Louis also, although he refuses to be ranked as a convert to the doctrines of M. Broussais, nevertheless admits the very great frequency of enteric follicular disease in fatal cases of continued fever, and he expresses his decided opposition to the precepts of his master, M. Chomel, who is the great champion in the present day of the essentiality of all fevers. In addition to these distinguished authors now mentioned, we may add a host

* With such an admission as this on the pathology of scurvy, is it not wonderful that Broussais and his disciples reject so dogmatically the influence of humoral vitiation in producing cholera and some forms of fever?

of others, as MM. Lallemand, Roche, Begin, Rayer, Duges, Billard, &c. all of whom are inclined to admit the correctness of Broussais' discoveries. M. Chomel himself in his recent work on Typhoid Fever, admits that "the follicular inflammation of the intestines is so frequent in those who have died from fever, that during the last five years of our practice, we have not met with a single contradictory example." But in spite of this admission, he still maintains, much to the dissatisfaction and displeasure of M. Bouillaud, the doctrine that fever is an idiopathic affection, and that the enteric disease is rather one of the effects, than the real cause, of the febrile action.

Such was the view which Laennec also took of the subject. His words are these: "the tumefaction of the mucous crypts or glands of Peyer, and the inflammation and ulceration of the intestinal mucous membrane are, in the majority of cases, evidently posterior to the development of the febrile symptoms, and consequently are no more the cause of these, than the cutaneous eruption is the cause of small-pox." The doctrine too of M. Broussais, that phthisis pulmonalis, scirrhus, and some other diseases of organic lesion are generally induced by chronic inflammation of the affected part, was vehemently opposed by M. Laennec, and after him by MM. Bayle and Louis. Even M. Bouillaud himself is too shrewd to commit himself to an unqualified admission of his master's doctrines on this point

But this is a subject which we cannot pursue at present, and which indeed has been only incidentally introduced by M. Bouillaud. The paramount object of our author is, to prove the local origin of continued fever, and the superiority of vigorous antiphlogistic remedies over every other mode of treatment in all febrile and inflammatory diseases. For this purpose, he appeals with confidence to the statistical tables of mortality from these diseases published by his opponents, and to the tables drawn up from his own practice at the great hospital La Charité.

We shall briefly notice these in succession.

M. Chomel in his Leçons sur la Fièvre Typhoide, Paris, 1834, estimates the average mortality, from this disease, at somewhat higher than one in every three cases. Out of 138 cases, which occurred in the wards of La Charité from 1822 to 1827, there were 50 deaths; of 18 cases in 1828 there were 5 deaths, and of 51 cases at the Hôtel Dieu, during the session of 1831-2, 16 proved fatal. The sum total therefore exhibits a mortality of 71 in 207 cases.

M. Louis in an Essay published by him in the Archives Generales for 1828, on Typhoid Fever, gave nearly the same estimate of its mortality; and in his recent work, Recherches sur la Fievre, &c. he furnishes a table of 150 cases; of these, 52 were fatal.

The results of M. Bouillaud's practice have been, according to his own report, much more successful. During the last three years he has treated 178* cases of typhoid fever, and of this number 22 only died—thus affording a proportion of only one in every eight. The average number of bleed-

* This is certainly a very large number of fever cases to be admitted into one of the Paris hospitals in the course of three years; but our author, who appears to have been sensible that some of his readers might suspect his accuracy, states that, being physician to the central bureau of admissions at the time, he was able to direct a great number of the fever cases to his own hospital, La Charité.

ings practised in these cases may be stated at three; and the quantity usually drawn at each time was four palettes or 16 ounces. In almost all the cases, blood was taken also by cupping or leeches, or by both. The other remedies were blisters and the internal use of the chlorurets.

M. Bouillaud is quite in raptures at his extraordinary success, and breaks out into the most exulting language at the thousands and tens of thousands of lives, which might be saved annually, if physicians were universally to adopt his antiphlogistic treatment.

We fear much that his hopes and expectations are far too sanguine. He may captivate and draw along with him a number of young students, who follow his clinique. His manner and the "tout ensemble" of his practice is quite calculated to gain the favour of the inexperienced.

We do not wonder at this; for we well recollect the time, when we ourselves used to be delighted, aye too and convinced, by the lectures of certain teachers, who took the same apparently simple views of many diseases, which M. Bouillaud inculcates; and who could see nothing in fevers but local inflammation of certain organ or organs, and whose practice therefore might be summed up in these words; bleed—bleed—bleed. Not so now with us, after experience has opened our eyes and matured our judgments.

If there is one position in medical practice, of the truth of which we are more thoroughly convinced than of another, it is this; that the character and type of epidemic diseases is not uniform and alike in all seasons; and therefore that a plan of treatment which may be very appropriate and successful during one epidemic, may be most pernicious during another.

No physician paid more attention to this subject—the difference in type of certain diseases in different seasons—that the English Hippocrates, Sydenham;* and all the best writers and most experienced practitioners of

---

* The following passage from Sydenham's great work is so full of sound practical wisdom, that we make no scruple to extract it entire. "Hoc saltem pro comperto habeo, ex multiplici accuratissimarum observationum fide, prædictas morborum species, præsertim febres continuas, ita toto, ut aiunt, cœlo differe, ut quâ methodo currente *anno* ægrotos liberaveris, eâdem ipsâ *anno jam vertente* forsitan e medio tolles; quodque, ubi semel in genuinam medendi rationem, quam hæc vel illa febris species vindicat, suspicato inciderim, ad eundem scopum collimans (favente, ut fit, optimo numine) metam quasi semper attingam; donec extinctâ illâ specie, novoque gliscente malo, anceps rursum hæreo, quâ mihi viâ insistendum ut ægris subveniam, ac proinde, nisi ingenti adhibitâ cautela intentisque omnibus animi nervis, vix ac ne vix quidem possum efficere, ne unus aut alter eorum, qui se primi meæ curæ commiserint, vitâ pericletetur, donec investigato jugiter, tandemque perspecto morbi genio ad eundem perdomandum recto pede et intrepidus denuo procedam. Variæ enim sunt annorum constitutiones, quæ neque calori, neque frigori, non sicco, humidove ortum suum debent, sed ab occultâ potius et inexplicabili quâdam alteratione in ipsis terræ visceribus pendent."

The Broussaian school, of which M. Bouillaud is so ardent and so enlightened a disciple, is unwilling to admit that diseases are influenced by such causes, as those alluded to in the preceding quotation. According to their creed, fevers are almost uniformly of the same type or character; always depending, as they assert, on the same morbid lesions of structures and requiring nearly the same mode of treatment. This is one of the most prominent errors of the physiological school.

this country, in London, Edinburgh, and Dublin, admit the accuracy of the statement. In Edinburgh especially, the character of the common epidemic, or typhus fever, has been most strikingly different at one time, from what it had been during a previous year.

In 1817-8 the prevailing fever required copious bleeding, and a strict antiphlogistic regimen; whereas, during the last few years, the leading physicians in the northern metropolis have, in most cases, not only abstained from depletions of blood, but have most successfully employed wine and other stimulants during the early stage. But we must now leave the subject of typhus fever,—the entero-mesenteritis of M. Bouillaud—and glance at some observations which our author has made on the treatment of one or two other diseases: and first of acute rheumatism. No one will deny that almost every case of this malady requires sanguineous depletion to some extent or another; but few will venture so far, as to talk of "strangling" it by large and repeated venæsections, as our author does. M. Bouillaud indeed seems to regard acute rheumatism as solely depending upon a vehemently excited state of the circulation, and to suppose that we have only to withdraw a certain portion of blood, and the morbid excitation will, as a matter of course, be quickly removed. This doctrine is full of error and danger.

There are numerous cases of acute rheumatism, in which, in spite of the most copious and quickly repeated bloodlettings, the symptoms will remain severe for eight, ten, or twelve days, and even then the patient is very far removed from a state of convalescence. Of all the tribe of the phlegmasiæ, there is none in which the blood itself is more seriously altered from a healthy state, than in acute rheumatism. If this be true—and the proofs are so numerous and well known to every experienced physician, that it is unnecessary at present to enlarge upon them—we are bound to consider the disease as affecting, not only the solids, but also the fluids—in short, to regard rheumatism as *partly*, and to a certain degree, a *humoral* disease. The blood itself has undergone certain changes, the most important, perhaps, of which seems to be a great and disproportionate increase in the quantity of the fibrinous matter; and, therefore, it cannot be by merely removing a portion of the circulating blood, that we should expect that a cure is to be effected. The invasion of acute rheumatism is seldom or never very sudden; it is usually preceded for a week or two, or even longer time, by certain premonitory symptoms, which the practised physician recognizes immediately, and by counteracting which in season, he is often enabled to anticipate, as it were, the threatening foe, and arrest him before he has gathered all his strength for the attack. This is the only rational way to "juguler" (we use M. Bouillaud's phrase) the disease. Blood must be taken to prevent the onset, or to check the progress of the synochal fever, which is always very violent in acute rheumatism; some form of mercurial preparation should be given to induce a slight affection of the gums, and hydragogue cathartics are to be administered, to act vigorously on the bowels and kidneys.

In no disease is the urine generally more scanty, deep-coloured, and loaded with urea and uric acid: it is often as dark and as thick as porter; and one of the earliest and most certain signs of the decrease of the disease, is the return of the urine to a healthy state, in quantity as well as in quality.* To promote this important object, alkalis, combined with purgatives and with colchicum, are by far the most potent means. Bouillaud takes no notice of this, or indeed of any other indication, except that of subduing the synochal excitement by drawing blood; and as for remedies, we hear of none, save "saignées," "sangsues," and "ventouses scarifiées."

He boastfully tells us that, of 101 cases which he has treated, he has lost only one; and the cause of death in that was from an extensive erysipelas. There is nothing wonderful in all this; for every one knows that a fatal case of acute rheumatism is one of the rarest events in medical practice.†

Differing, as we do, from M. Bouillaud, as respects the etiology, and in part also the treatment, of acute rheumatism, it is but proper that we should state, that the average quantity of blood, which he draws in violent cases of this disease, is not much greater than we should feel inclined ourselves to do. He states it at about five pounds; this is rather too much. If we *must* state a quantity, we should say between three and four pounds.

The urgency, with which our author explains and inculcates his practice, affords to our minds satisfactory evidence, that the prevailing mode in France of treating acute rheumatism is very feeble and dilatory;‡ and we are, therefore, the more willing to judge favourably of his writings, as they may pos-

---

* For further information on this interesting and very important subject of practical medicine, the attention of the reader may be directed with advantage to one of the articles in the foreign periscope of this Review for last July, "on the Urine, as affording Indications in the Treatment of certain Diseases."

† It is amusing to observe with what pertinacity of opposition our author pursues M. Chomel, and strives, on all occasions, to expose his errors and inconsistencies. He has accordingly hunted out a case of acute rheumatism, in which death took place, reported in the Bulletin de Therapeutique, for April 1836, from the clinique of M. C. at the Hôtel Dieu. The anonymous contributor of the case had insinuated that its fatal issue had been accelerated by the depletory practice, which had been followed. This might seem to militate against the doctrines of M. Bouillaud; but he very justly remarks in reply, that the frequency and extent of the bleedings in the reported case greatly exceeded what he himself has ever recommended or adopted. Instead of three or four bleedings, each of from 12 to 15 ounces, the poor patient, a lymphatic girl, was actually bled eight different times, and the quantity of blood drawn at each time varied from 18 to 30 ounces!!

This is out-Heroding Herod in grand style! Such is an example, and we might adduce a hundred somewhat similar, of the rash, unscientific, and most irrational practice of some French physicians. There is that strong tendency to *outrance* in every thing they do, that we sober folks on this side of the Channel can never place much confidence in their practical precepts.

‡ We find that our suspicions are quite correct. On examining the article "Rhumatisme," in the Dictionnaire de Médécine, 1827, the treatment there recommended cannot be too much condemned for its inertness. Mentioning venæsection as one of the remedies in acute rheumatism, the writer says :—"Toutefois il est peu de phlegmasies, où les indications de ce moyen sont plus rares qu'ici. L'avantage des evacuations sanguines locales est, au contraire, généralement reconnu dans la maladie qui nous occupe." The rest of the precepts are all of the same stamp. Not even any reference is made to the use of calomel, of colchicum, or of purgatives. The whole article, in short, is one of the very worst, which we ever met with in any scientific dictionary.

sibly have the effect of introducing a more active and energetic system of therapeutics among his countrymen.

That M. Bouillaud is far too much of a dogmatist, and far too little of a rational empiric, in his doctrines and in his practice, is very apparent from the tone of his remarks on every subject, which he treats. Occasional occurrences speedily assume with him all the importance of constant and characteristic phenomena. We have already seen how stoutly he maintains the universality of intestinal mucous disease in all fevers; and equally bold and stubborn is he in asserting, that cardiac inflammation, in the form of pericarditis or of endocarditis, is an almost uniform complication in acute rheumatism. But we must now quit this subject, and briefly allude, before drawing the article to a close, to the results of French practice in another most frequent form of phlegmasia—pneumonia.

In the article, pneumonia, in the Nouveau Dictionnaire de Medecine, M. Chomel estimates the average mortality of this disease in hospital practice, at one fourth of all the cases. M. Louis has rated it as high as a third of the whole. Of ninety cases treated by M. Gueneau at the Hôtel Dieu in 1829, 38 died. Of 63 treated by M. Bertin at the Hospital Cochin in 1822, 16 proved fatal; and of 24 cases reported from the Clinique of M. Cayol at the Charité, 6 were fatal.

M. Laennec, in treating of the effects of tartar emetic in pneumonia, alludes to his great success with it in these words:—

"In 1824 I treated with the tartar emetic 28 cases of pneumonia, either simple, or complicated with a slight pleuritic effusion. All the patients, with the exception of one, 70 years of age, and who was not able to *tolerate* the remedy, were cured, although in most the disease was very serious. In 1826, I have treated 34 cases. Five of these have proved fatal; but it is to be noticed that in two of the fatal cases, the patients, both advanced in life, were moribund, when brought into the hospital; in the third, the pneumonia was complicated with heart disease; the fourth proved fatal from chronic pleurisy, while the patient was recovering from pneumonia; and in the fifth, there was a co-existent cerebral congestion in an old man of 72."

These results of M. Laennec's practice appear indeed very flattering; but M. Bouillaud confesses himself quite perplexed to explain the disagreement between them and the results of other tables, which have been published by M.M. Lagarde and Leconteulx, both attached formerly to the service of M. Laennec.

The former gentleman has given the reports of sixteen cases which were treated by the above, the antimonial, method under the care of M. Laennec, and of these five proved fatal; and M. Leconteulx has added the reports of other 30 cases treated by M. Laennec during the years 1825 and 1826: twelve of these patients died. Let us now attend to the results of M. Bouillaud's own practice. He has had 152 cases of pleuro-pneumonia under his care, from September 1831, to March 1836: of these, 18 only have been fatal.

The mortality, therefore, has been rather less than an eighth of the whole—a very striking difference from that furnished by the clinique of other hospital physicians.

We cannot afford space for the various tables, which M. Bouillaud has published, and shall therefore only mention a few of the particulars. The average number of bleedings was four, the quantity drawn at each being 16 ounces; of cuppings one, the quantity drawn being 12 ounces; of leeches 30 or rather more. It is to be noticed, that all this drawing of blood in various ways, took place within the first three or four days of the treatment; and M. Bouillaud very justly remarks that double the quantity, taken at longer intervals,—a practice too generally followed by his countrymen—would not have the same efficacy. The average duration of the disease, before convalescence had commenced, is stated by our author at ten days.

In perusing attentively the different tables, which M. Bouillaud has published in support of his views, the English critic cannot fail to be struck with the indiscriminate character of many of his statements, and of not a few of his precepts. For example, he finds that, in very many cases, large and repeated bleedings were practised, even when the pneumonic inflammation had reached its third period, or stage—that of infiltration. Such practice, we need scarcely say, is most dangerous and improper. When the disease has been allowed to proceed so far, the prognosis must always be very unfavorable; and most certainly the chances of the patient's recovery will not be enhanced by the "jugulante" treatment, which M. Bouillaud recommends. The moderate use of blood-letting may be, we do not deny, still necessary; but only with the greatest care and caution. The topical abstraction of blood by cupping, the application of blisters, the exhibition of small doses of mercury with squills and such like medicines—these are the remedies which a British physician would have recourse to against pneumonia in the third stage.

But we have had so often an occasion of late years to express, in the pages of this Journal, our disapproval of M. Bouillaud's therapeutic precepts, that it is quite unnecessary to enlarge upon this topic at present. His work on Medical Philosophy has only tended to confirm the opinions which we had previously formed of his character as a physician—ardent, clever, and indefatigable; but rash, rapid, and far too partial. In truth there is very little genuine reasoning or induction in it, and the whole leaves the impression of its being rather an ingenious, though disproportionately lengthened, Essay for a debating society, than a philosophical treatise for study in the closet.

